# Alpha-7 nicotinic acetylcholine receptor agonist alleviates psoriasis-like inflammation through inhibition of the STAT3 and NF-κB signaling pathway

**DOI:** 10.1038/s41420-022-00943-4

**Published:** 2022-03-30

**Authors:** Yiwen Chen, Panpan Lian, Ziqi Peng, Junaid Wazir, Chujun Ma, Lulu Wei, Li Li, Jun Liu, Chen Zhao, Wenyuan Pu, Hongwei Wang, Zhonglan Su

**Affiliations:** 1 Department of Dermatology, The First Affiliated Hospital of Nanjing Medical University, Nanjing, 210029 P.R. China; 2 State Key Laboratory of Analytical Chemistry for Life Science & Jiangsu Key Laboratory of Molecular Medicine, Medical School of Nanjing University, Nanjing, 210093 P.R. China; 3 Department of Dermatology, Drum Tower Hospital, Medical School of Nanjing University, Nanjing, 210008 Nanjing, P.R. China

**Keywords:** Neuroimmunology, Immunological disorders, Psoriasis

## Abstract

Psoriasis is a chronic inflammatory cutaneous disease; it has been discovered that stimulation of the nervous system increases susceptibility to psoriasis. Although the cholinergic anti-inflammatory pathway, which is mediated by the alpha-7 nicotinic acetylcholine receptor (α7nAChR), is critical for controlling multiple types of inflammation, its expression pattern and pathogenesis function in psoriatic lesioned skin tissue are unknown. We hereby analyzed the expression of α7nAchR in human and mouse psoriatic skin tissue. In vivo, PNU-282987 or Methyllycaconitine, a specific agonist or antagonist of α7nAchR, were administered to imiquimod (IMQ)-induced psoriatic mouse models. The macroscopic appearance and histopathological features of the psoriatic mice skin were evaluated. In addition, cell proliferation and differentiation markers were investigated. The level of pro-inflammatory cytokines released from the lesioned skin, as well as the activation of the relevant signaling pathways, were measured. Our findings indicated that psoriatic lesional skin expressed an increased level of α7nAChR, with its tissue distribution being primarily in skin keratinocytes and macrophages. In an IMQ-induced murine psoriasis model, α7nAChR agonist PNU-282987 treatment alleviated psoriasis-like inflammation by down-regulating the expression of multiple types of pro-inflammatory mediators and normalized keratinocyte proliferation and differentiation, whereas α7nAChR antagonist treatment exacerbated its effect. Mechanically, we observed that activation of the α7nAChR inhibited the activation of the STAT3 and NF-κB signaling pathways in in vitro cultured HaCaT cells induced by Th17-related cytokine IL-6/IL-22 or Th1-related cytokine TNF-α. Taken together, these findings demonstrate that attenuation of psoriatic inflammation via the cholinergic anti-inflammatory pathway is dependent on α7nAChR activation.

## Introduction

Psoriasis is a chronic and recurrent inflammatory disease, affecting around 2–3% of the population worldwide [1]. The major pathological features of psoriasis include epidermal hyperplasia, hyperkeratosis, and skin inflammation. The immune pathogenesis of psoriasis is still unknown, however current research suggests that the interaction between keratinocytes and immune cells is crucial [2]. The activation of infiltrated immune cells releases multiple types of pro-inflammatory factors that trigger the abnormal differentiation and hyper-proliferation of skin keratinocytes, accompanied by the increased formation of small blood vessels [3].

Although the exact cause of psoriasis is unknown, it is widely believed that both genetic predisposition and environmental factors influence the disease’s development [4]. The identified risk factors that have been reported to exacerbate the disease manifestations include traumatic injury to the skin, physical and psychological stress, nutrition status, infections, weather, excessive alcohol intake, and drugs such as lithium and β-blockers [5]. Importantly, there is mounting evidence that abnormal nervous system stimulation is linked to psoriasis susceptibility. During this process, multiple neurons associated molecules, such as neuropeptides, nerve growth factors, and various types of neurotransmitters, contribute to the immune-pathological mechanism [6].

Acetylcholine is the major neurotransmitter of the parasympathetic nervous system, which regulates smooth muscles, blood vessels, bodily secretions, and heart rate. A recent study reported that vagal nerve stimulation can attenuate systemic inflammation through the cholinergic anti-inflammatory pathway (CAP), with alpha-7 nicotinic acetylcholine receptor (α7nAchR) being an essential regulator [7]. Acetylcholine (ACh) secreted from the vagus nerve could suppress cytokine production and macrophage activation via interaction with α7nAChR on neurons and inflammatory cells. The activation of α7nAChR by ACh or other specific agonists has been reported to decrease the expression of pro-inflammatory cytokines in several types of immune cells. However, whether CAP could regulate the pathological development of psoriasis is still a question that needs to be defined.

In this study, we found that α7nAChR was mostly expressed in epidermal keratinocytes in human skin. Skin specimens from clinical psoriatic patients or animal models had higher levels of α7nAChR in lesioned skin epidermis, indicating that the nervous system is involved in the pathogenesis of psoriasis. PNU-282987, a selective agonist of α7nAChR [8], when administrated in vivo, could ameliorate the psoriatic skin lesions in IMQ-induced psoriatic inflammation, which includes alleviating the erythema, scales, and infiltration of inflammatory cells. The abnormal proliferation and differentiation of keratinocytes in psoriatic lesion was significantly inhibited in response to PNU-282987 treatment, the pro-inflammatory cytokines of IL-1β, IL-6, and TNF-α in skin lesion were decreased after PNU-282987 treatment in vivo and in vitro. Mechanistic study indicated that the protective role of PNU-282987 in psoriasis was attributed to the suppression of STAT3 and NF-κB signal pathways. In a psoriatic mouse model, treatment with the α7nAChR antagonist Methyllycaconitine citrate worsened the cutaneous inflammation.

## Results

### Increased expression of α7 nicotinic acetylcholine receptor was detected in the human and mouse psoriatic lesional skin tissue

**Fig. 1:**
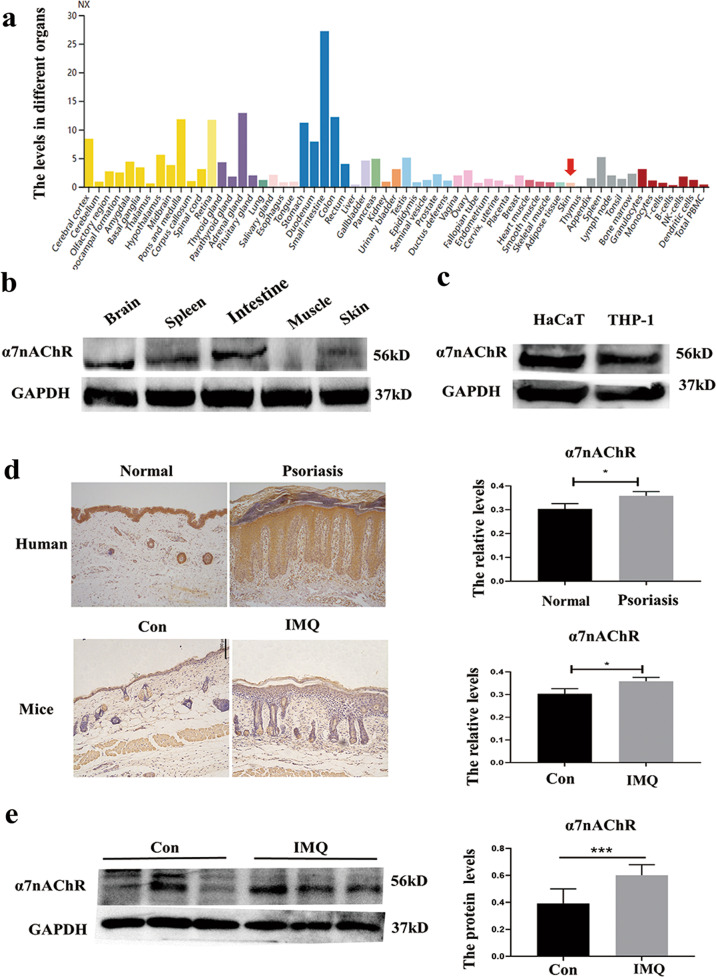
Increased expression of α7 nicotinic acetylcholine receptor was detected in the human and mouse psoriatic lesional skin tissue. **A** The protein and mRNA of α7nAChR were expressed in skin tissue (arrows) from the Human Protein Atlas website. The red arrow indicates the levels of α7nAChR in skin tissue. Upper panel: protein levels, lower panel: mRNA levels. **B** Immunohistochemical staining indicated that α7nAChR was distributed throughout the human epidermis of skin from a healthy individual and psoriasis patient (magnification ×100). **C** Western Blot assay confirmed the expression of α7nAChR in HaCaT cells compared to THP-1 cells. **D** Immunohistochemical staining of α7nAChR in skin from control group and IMQ-induced model group (magnification ×100; *n* = 6). **E** Analysis of the expression levels of α7nAChR protein in different organs, including brain, spleen, intestine, muscle, and skin from BALB/c mice. GAPDH was used as the internal control.

To determine α7nAChR gene expression across different tissues, we first analyzed the mRNA expression of α7nAChR using the RNASeqV2 database available from the open access “the Human Protein Atlas portal website (http://www.proteinatlas.org/)”. According to the mRNA expression database, there was positive expression of α7nAChR in human skin tissue, and the level of α7nAChR expression in human skin was intermediate when compared to other organs or tissues (Fig. 1A). To confirm this result, different organs including the brain, spleen, intestine, muscle, and skin were harvested from BALB/c mice and subjected to the western blot assay. Our results indicated that the brain, spleen, and intestine had relatively high levels of α7nAChR expression, whereas the skin and skeletal muscle had intermediate or low levels of α7nAChR expression (Fig. 1B). These results indicated that the expression pattern of α7nAChR in humans and mice seems to be identical. To explore whether α7nAChR is expressed in keratinocytes, we compared the expression of α7nAChR in HaCaT cells, an immortalized human keratinocyte line, to THP-1 cells, a human monocytic cell line derived from an acute monocytic leukemia patient that has been reported to positively express α7nAChR [9], thus were used as positive control. Our findings confirmed the protein expression of α7nAChR in skin keratinocytes (Fig. 1C). Next, we used immunohistochemistry (IHC) staining to determine the change in expression of α7nAChR in skin samples from psoriasis patients and healthy volunteers. The results showed that the expression of α7nAChR was significantly increased, which was mainly distributed in the epidermal layer in the human skin. Additionally, this pattern of expression was confirmed in the skin of IMQ-induced mice (Fig. 1D). The western blot assay also revealed that IMQ-induced psoriatic lesions had higher levels of α7nAChR. (Fig. 1E).

### In vivo administration of α7nAChR agonist alleviated inflammatory skin lesions in an IMQ-induced psoriatic mouse model

**Fig. 2:**
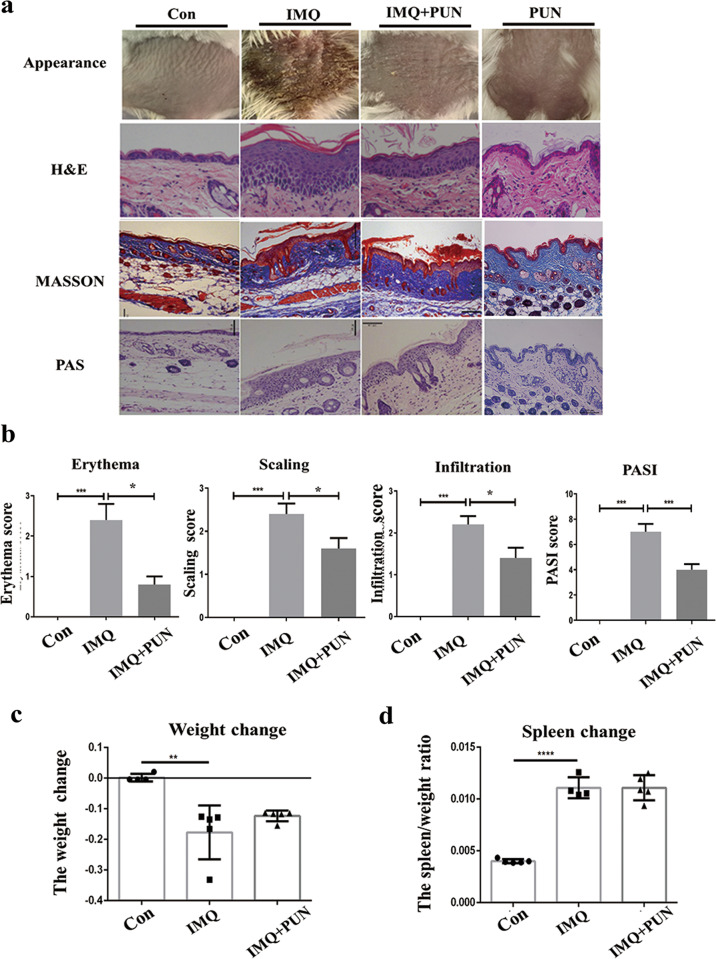
α7nAChR activation ameliorates the psoriatic skin lesions in IMQ-induced mice. PNU-282987, a selective agonist of α7nAChR, counteracted the psoriasiform lesion in IMQ-induced mice. **A** H&E staining and epidermal thickness measurement were analyzed in a different group (magnification ×100). **B** Erythema, scaling, infiltration, and PASI score indicated IMQ + PNU-282987 group had improved symptoms than the IMQ group. Data were presented as mean ± SD, *n* = 6. **C** Weight change to weight ratio was at a low level in IMQ-induced model group. **D** Spleen change to weight ratio was calculated to analysis the inflammatory state in mice. Data are presented as mean ± SD, *n* = 6. *P < 0.05, **P < 0.01, ***P < 0.001.

To assess the function of α7nAChR in vivo, IMQ-induced psoriasis-like skin inflammation model was used by consecutive topical application of IMQ (62.5 mg each day) on the dorsal skin of mice, following the methods as previously described. To evaluate the effect of CAP on IMQ-induced psoriasis, PNU-282987 (PNU-282987), a selective α7nAChR agonist, was injected intraperitoneally (10 mg/kg) 1 h prior to the daily IMQ treatment for 6 days. As shown in Fig. 2A, the macroscopic appearance of the skin lesions was significantly relieved by PNU-282987 treatment, with reduced scaling, erythema, and infiltration in the PNU treatment group compared to the IMQ psoriatic model control. PNU-282987 treatment resulted in a significant reduction in the PASI score (Fig. 2B), H&E staining of skin biopsy samples confirmed that PNU-282987 treatment ameliorated the pathological epidermodysplasia phenotype, including epidermal thickness and immune cell infiltration. Masson staining and PAS staining failed to detect any positive signaling which further confirmed that the collagen deposition and glycogen accumulation were not involved in the pathological development of experimental psoriasis (Fig. 2A). Moreover, the body weight loss, together with the enlarger spleen in IMQ-induced psoriatic mice, were not restored after PNU-282987 treatment (Fig. 2C, D).

### In vivo, treatment with a α7nAChR agonist inhibited the inflammatory response and prevented the abnormal differentiation of epidemic cells in psoriatic mice

**Fig. 3:**
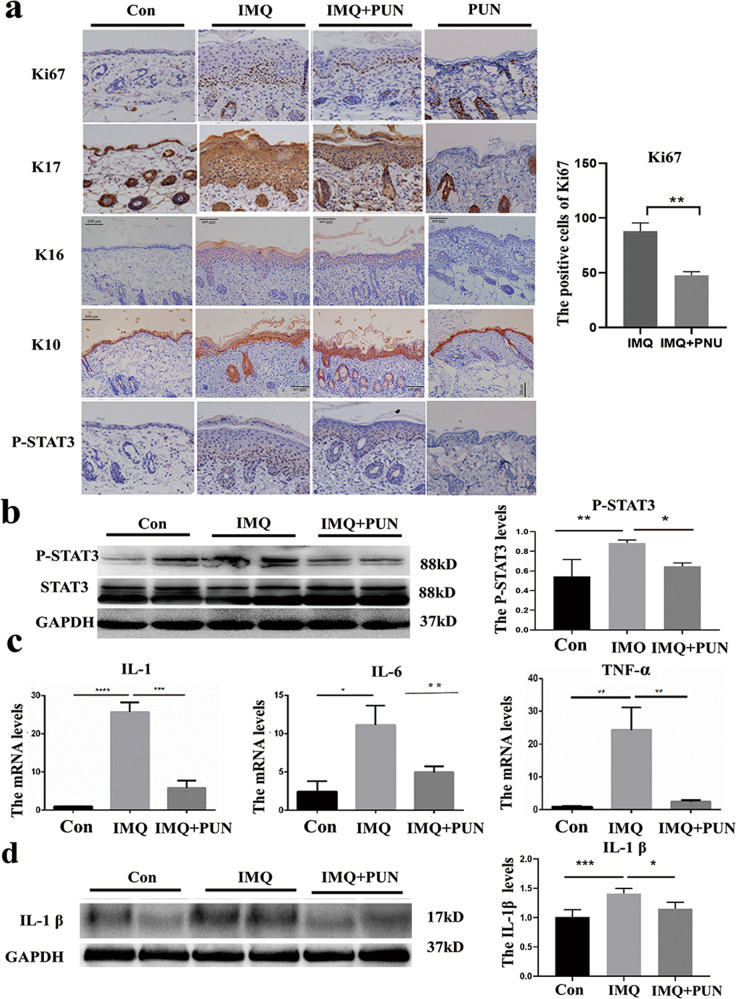
PNU counteracts inflammatory response and abnormal differentiation of epidemical cells in vivo and in vitro. **a** Ki67, K17, K16, K10, and P-STAT3 immunohistochemical staining in lesion skin of IMQ-induced mice (magnification × 100). **b** The relevant proteins of STAT3 and P-STAT3 in lesion skin were measured by western analysis. **c** The mRNA levels of IL-1β, IL-6, and TNF-α in mouse skin lesions were measured by the qPCR assay. **d** The protein levels of IL-1β in lesions skin were determined by western blot. The values are normalized to GAPDH. *n* = 6. *P < 0.05, **P < 0.01, ***P < 0.001.

In the IMQ-induced psoriatic mouse model, the histological analysis showed that PNU-282987 treatment could reduce skin thickness and inhibit epidermal proliferation. To explore its molecular basis, the growth dynamics of epidemical cells in mice was assessed based on the expression level of Ki67, a marker indicating proliferative keratinocytes, keratin 17 (K17) and keratin 16 (K16), an intermediate differentiation marker that linked with the abnormal differentiation of keratinocyte, as well as keratin 10 (K10) a normal marker of skin differentiation [10]. Our result showed that the PNU-282987 treatment significantly decreased abnormal proliferation and differentiation of epidemical keratinocytes (Fig. 3A). STAT3, a transcription factor, has been shown to control cell cycling and proliferation in keratinocytes, making it a critical signaling pathway involved in the pathological development of psoriasis [11]. IHC staining showed that there is aberrant activation of STAT3 in psoriatic mouse skin. Active STAT3 signaling was detected primarily in the epidermis’s basal layer in lesioned skin, and PNU-282987 administration reduced STAT3 activation in lesioned skin of IMQ mice (Fig. 3A). Additionally, there was a significant decrease in P-STAT3 expression in lesioned skin of the PNU-282987 treatment group compared to the IMQ-induced psoriatic model group, as determined by western blot analysis (Fig. 3B). Given that chronic inflammation is a hallmark of psoriasis, we then perform an RT-PCR assay to determine the mRNA levels of IL-1β, IL-6, and TNF-α. As expected, PNU-282987 treatment significantly decreased the expression of these pro-inflammatory cytokines at the mRNA level, implying that PUN treatment has a protective effect in terms of inflammation attenuation (Fig. 3C). To corroborate these findings, the reduced expression of IL-1β was also detected using a western blot assay (Fig. 3D).

### In vitro administration of α7nAChR agonist restore Th17 cytokines induced keratinocyte over proliferation and abnormal differentiation by inhibiting STAT3 signal pathway

**Fig. 4:**
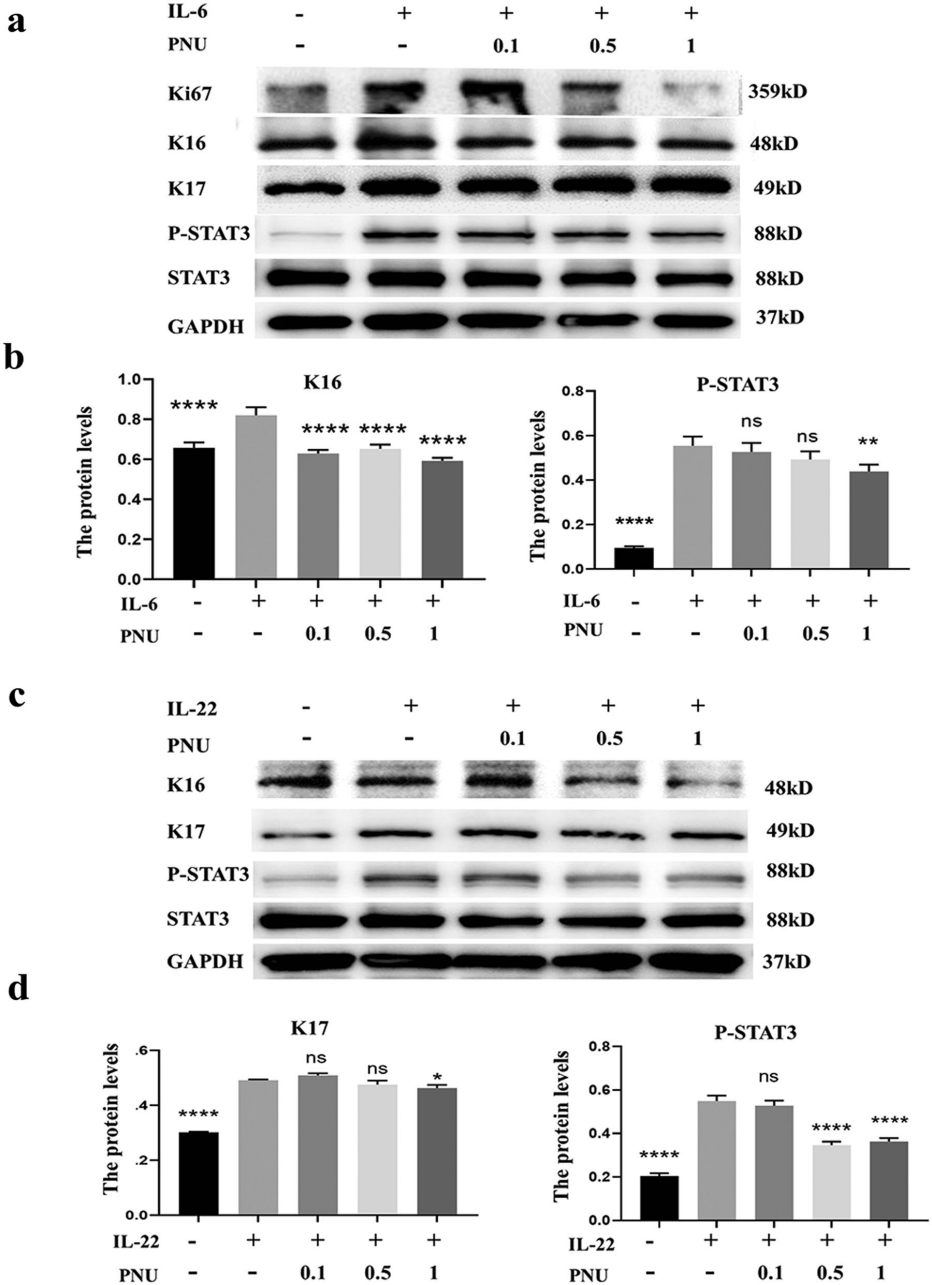
PNU-282987 counteracts IL-6/IL-22 induced cutaneous inflammation and promotes differentiation by inhibiting STAT3 signaling pathway. **a** HaCaTs were pretreated with different concentrations of PNU-282987 (0.1 uM, 0.5 uM, 1 uM) for 12 h followed by stimulation of 25 ng/ml IL-6 or IL-22 for 30 min. The expression of Ki67, K16, K17, P-STAT3, and STAT3 were determined by western blot in response to IL-6 (**a**) or IL-22 (**c**) treatment. The values are normalized to GAPDH. The activation of STAT3 signaling pathway and its related proteins expression were quantified with the intensity of bands on Western blot by image J and Graphpad Prism software (**b**, **d**). *P < 0.05, **P < 0.01, ***P < 0.001.

As it is widely accepted that Th17 immune responses contribute to psoriatic inflammatory responses, we investigated the role of α7nAChR activation in the activation of Th17-related signaling pathways. IL-6 and IL-22 are Th17-related cytokines that activate STAT3 synergistically and contribute to the pathological development of psoriasis [12]. Moreover, epidermal keratinocytes in psoriatic lesions are characterized by activated STAT3, and increased levels of cytokines and growth factors that promote STAT3 activation have been found within psoriatic lesions. We found that the administration of 25 ng/ml IL-6 or IL-22 in HaCaT for 30 mins significantly upregulated the phosphorylation of STAT3 at Tyr705 site (Fig. 4A, C), which was consistent with previous results [14]. The activation of the α7nAChR by its agonist PNU-282987 significantly inhibited the STAT3 signaling activation induced by IL-6 and IL-22 (Fig. 4B and D), indicating that PNU-282987 had the potential to inhibit Th17-mediated inflammation in the skin and alleviate the psoriasis symptom. Furthermore, PNU-282987 treatment reduced the expression of abnormal differentiation makers such as K17 and K16. We then measured cell proliferation using the proliferation marker Ki67 and found that α7nAChR activation could prevent keratinocyte over-proliferation induced by IL-6 or IL-22 (Fig. 4A, C).

**Fig. 5:**
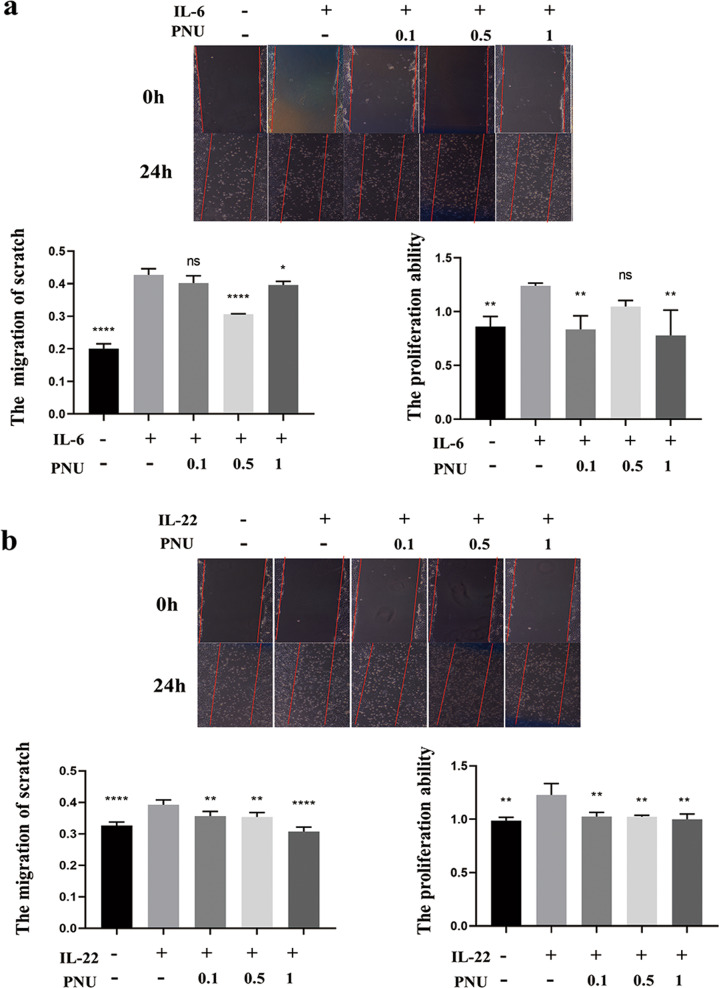
PNU-282987 treatment inhibits the IL-6 or IL-22 induced keratinocyte migration. The effect of PNU-282987 treatment on IL-6 (**a**) or IL-22 (**b**) induced cell migration was analyzed by in vitro scratch‐wound assay, which were established in IL-6‐treated and untreated HaCaT cells. Scratch‐wound assay result was analyzed and the percentage of closed wound area is presented in the graph.

After demonstrating that α7nAChR activation had an antagonistic effect on the STAT3 signaling pathway, we used an in vitro wound scratch healing assay to investigate the effect of α7nAChR activation on IL-6 or IL-22 stimulated wound healing. As expected, IL-6 or IL-22 treatment significantly increased the wound healing rate; however, upon PNU treatment, the average wound healing rate was significantly down-regulated (Fig. 5A, B), and both the average migration rate and the cell proliferation level were significantly inhibited, indicating the anti-proliferation effects of PNU-282987 treatment.

### In vitro administration of α7nAChR agonist reduced TNF-α induced inflammatory response in keratinocytes by inhibiting NF-κB pathway

**Fig. 6:**
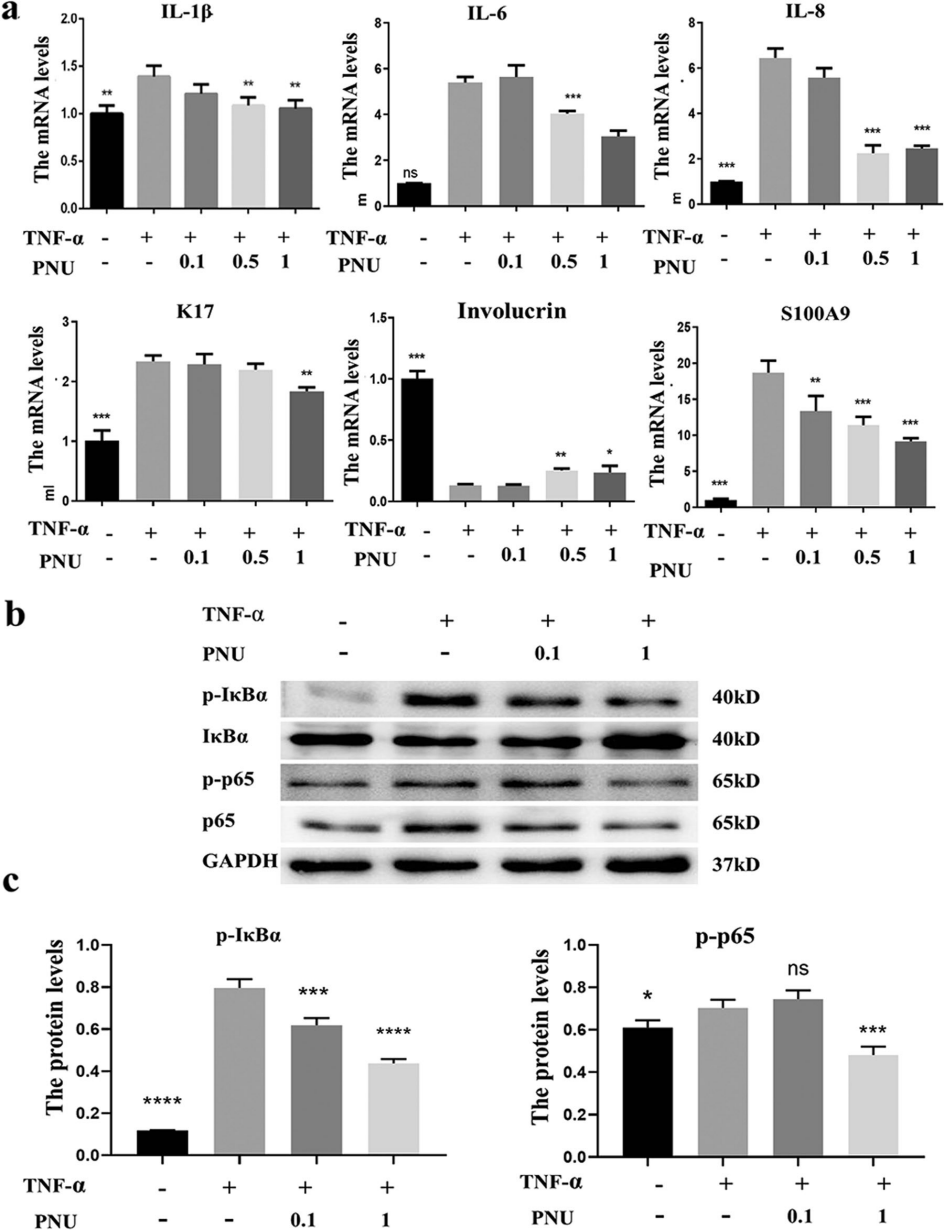
PNU-282987 counteracts TNF-α induced cutaneous inflammation by inhibiting NF-kB signal pathway. **a** HaCaTs were pretreated with different concentrations of PNU-282987 (0.1 uM, 0.5 uM, 1 uM) for 12 h followed by stimulation of TNF-α (20 ng/ml) for 24 h, the transcriptional expression levels of K17, Involucrin, S100A9, IL-1β, IL-6, IL-8 were detected by RT-PCR. **b** The expression of p-IκBα, IκBα, p-p65, and p65 were determined by western blot in response to PNU-282987 (0.1 uM, 1 uM) and TNF-α (20 ng/ml) treatment. **c** The relative expression levels of P-IkB and P-P65 were analyzed by normalized to GAPDH.

Keratinocytes are important effector cells in psoriasis because their hyper-proliferation and abnormal differentiation contribute to the skin phenotype of silvery scale plaques [13]. TNF-α is thought to play a key role in the pathogenesis of psoriasis, and anti-TNF-α therapy has proven to be effective [14]. Therefore, we used TNF-α induced HaCaT cells as the psoriatic cell model to investigate the effect of α7nAChR agonist in psoriatic pathology. The expression profile of pro-inflammatory cytokines including IL-1β, IL-6, and IL-8 were significantly increased after exposure to TNF-α (20 ng/ml). PNU-282987 treatment, dose dependently (0.1 uM, 0.5 uM, 1 uM), suppressed the expression of IL-1β, IL-6, and IL-8 at mRNA level (Fig. 6A). Consistent with this finding, the keratinocyte differentiation factors K16, K17, Involucrin, and S100A9 were also restored (Fig. 6A), indicating that PNU-282987 treatment reduced inflammatory factor expression and normalized keratinocyte differentiation in IMQ-induced mice or TNF-α stimulated HaCaT cells. Given that NF-κB is a critical regulator of the inflammatory response in a variety of diseases, we investigated whether the anti-inflammatory mechanism of PNU-282987 was mediated by inhibiting NF-κB pathway. It was evidenced that PNU-282987 administration inhibited IkB alpha and p65 activation (Fig. 6B, C), implying that PNU-282987 exerted its anti-inflammatory effects via inhibition of the NF-κB signaling pathway.

### Methyllycaconitine citrate, a selective α7nAChR antagonist, exacerbated the abnormal proliferation and differentiation in psoriatic mice model

Since our findings show that PNU-282987, a selective α7nAChR agonist, can improve the lesioned skin of IMQ-stimulated mice as well as IL-6/IL-22 induced HaCaT cells, we next investigate the effect of Methyllycaconitine citrate (METH), a selective α7nAChR antagonist, on IMQ-induced psoriasis.

**Fig. 7:**
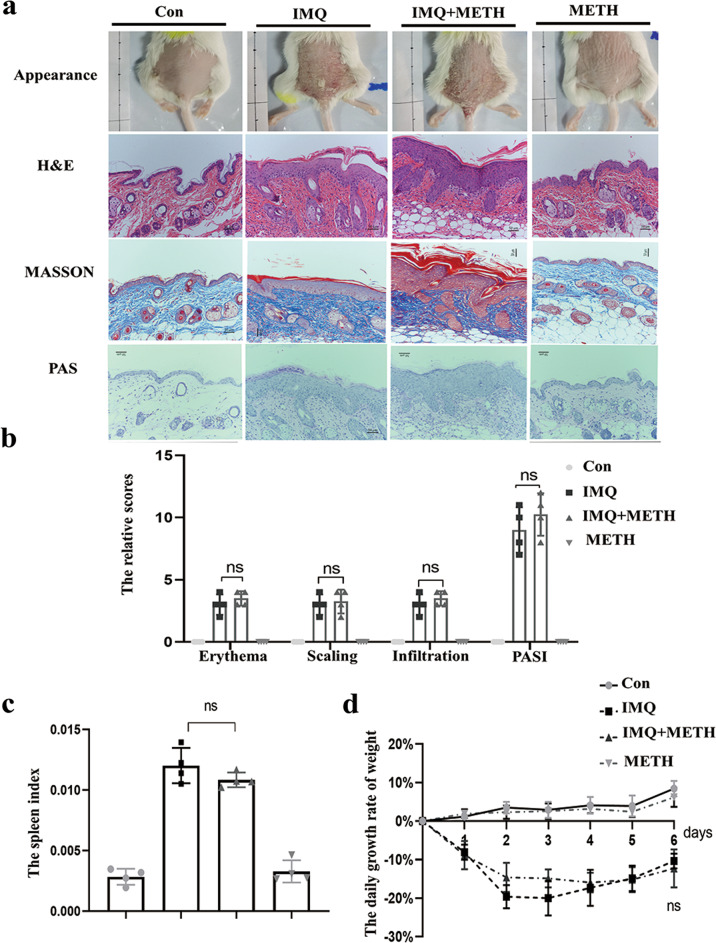
α7nAChR antagonist treatment exacerbates the abnormal epidermal proliferation and differentiation in psoriatic mice model. **a** The application ofα7nAChR antagonist Methyllycaconitine citrate led to mildly aggravated psoriasis. H&E staining and epidermal thickness measurements were analyzed in a different group (magnification ×200). **b** Erythema, scaling, infiltration, and PASI score indicated IMQ + METH group had slight worsen symptoms than the IMQ group, but there was no statistical difference. **c** Spleen change to weight ratio was calculated to analysis the inflammatory state in mice. **d** Mice in IMQ + METH group had the similar daily growth rate of weight to IMQ-induced model group. Data are presented as mean ± SD, *n* = 6.

**Fig. 8:**
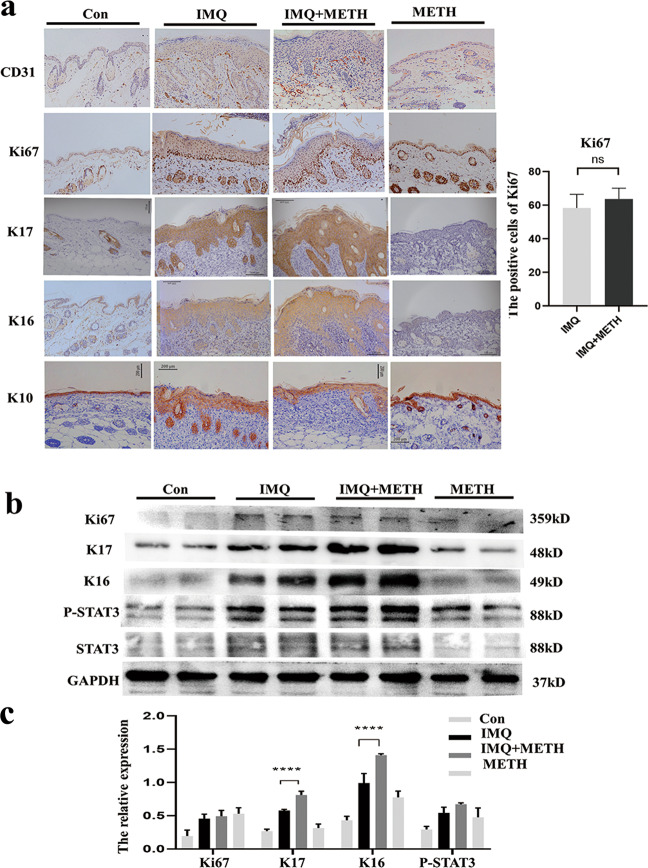
α7nAChR antagonist treatment exacerbates the cutaneous inflammation in IMQ-induced psoriatic mice model. **a** Immunohistochemical staining of CD31, Ki67, K17, K16, and K10 in lesional skin of IMQ-induced mice. (Arrow: the positive cells. magnification ×200). **b** The protein levels of Ki67, K17, K16, STAT3, and P-STAT3 in skin lesion were measured by western blot assay. **c** The relative protein levels were accessed by image J software.

The appearance of the back skin and H&E staining revealed that the mice group that received METH treatment had an increased pathological manifest in skin lesions and epidermal skin thickness (Fig. 7A). The score of erythema, scaling, and infiltration in the IMQ + METY group was slightly higher than in the IMQ-induced psoriatic group, but there was no statistical difference (Fig. 7B). Moreover, Masson and PAS staining showed that the pathological basis of psoriatic model was not accompanied by collagen deposition and glycogen accumulation (Fig. 7A). Besides, the spleen-to-weight ratio of the METH treatment group remained high as compared to the model group, indicating that systemic inflammation was in an abnormally active state (Fig. 7C). Furthermore, the daily weight growth rate in the IMQ + METH group was the same as in the IMQ group, with a first drop on days 2 and 3 and then an ascent (Fig. 7D). It is widely assumed that the formation of new blood vessels in the dermal layer was to be held responsible for the infiltration of multiple immune cells that began with early psoriatic changes. Our findings revealed that CD31, an angiogenesis marker, was more abundant and distributed more widely in the dermis of the IMQ + METH group (Fig. 8A). Although the appearance and PSAI score in IMQ + METH mice showed little change with no statistical difference, histological hallmarks of psoriatic skin, including Ki67, K17, K16, and K10 expression, revealed that Methyllycaconitine citrate application aggravated the dynamics of abnormal keratinocyte proliferation and differentiation (Fig. 8A). Additionally, the expression levels of K17, K16, and P-STAT3 in lesional skin were significantly increased after Methyllycaconitine citrate administration, particularly for K17 and K16 (Fig. 8B, C).

## Discussion

The CAP is important in the regulation of various types of inflammation, such as sepsis and asthma [15, 16]. Its role in inflammatory skin disorders, however, is largely unknown. In the present study, we investigated the protective role of CAP activation in the pathogenesis of psoriasis. We demonstrated that pharmacologically activating CAP with PNU-282987 reduced cutaneous inflammation in an IMQ-induced experimental psoriatic model, whereas CAP inhibitor Methyllycaconitine citrate had the opposite effect. The protective effects of PNU-282987 were characterized by a reduction in the release of inflammatory cytokines and the inhibition of inflammatory signaling pathways. Overall, our findings provide direct experimental evidence that the CAP is important in regulating the inflammatory response during experimental psoriasis.

The CAP is mediated by the vagus nerve, which releases acetylcholine to interact with the α7 subunit of the nicotinic acetylcholine receptor (α7nAChR) on multiple types of immunoregulatory cells. A recent study indicated that non-neuronal cells can also release acetylcholine and express the α7nAChR, which includes T cells, B cells, macrophages, fibroblasts, and keratinocytes [17]. In mouse skin, α7nAChR has been found expressed in mononuclear cells (MNCs) and fibroblastic cells (FBCs), including macrophages, fibrocytes, and myofibroblasts, and its expression is time-dependent, indicating that it is involved in distinct cell types during the process of skin wound healing in mice [18]. We first assessed the expression level of α7nAChR on human and mouse skin. The results indicated that, when compared to other organs or tissues, the relative expression level of α7nAChR was intermediate or high in the skin epidermis. Furthermore, the protein level expression of α7nAChR was detected in both macrophage and skin keratinocytes, indicating that its immune regulation function may be mediated via skin macrophage and keratinocytes.

Notably, data from our current study revealed that there was a significant increase in expression of α7nAChR in lesioned psoriatic skin when compared to healthy control skin or no lesioned skin. Given the well-known anti-inflammatory activity of CAP, we speculated that α7nAChR might play a role in the anti-inflammatory response and repair process during the psoriatic inflammatory disorder. The increased expression of this receptor during cutaneous inflammation may be a self-regulatory anti-inflammatory response that helps to maintain immune homeostasis and avoid tissue injury caused by an overwhelming inflammatory response.

As a chronic inflammatory skin disorder, psoriasis has been considered as a T cell-mediated autoimmune disease, with the interaction of antigen-presenting cells (APCs), T cell subsets, and skin keratinocytes playing an important role in the pathological development of psoriasis. T cell-related immune responses, especially Th17 and Th1 immune responses, are important in the pathological development of psoriasis. Th1 type immune responses are thought to contribute to the initiation stage of psoriasis inflammation [19], whereas Th17 dominated immune responses are thought to be responsible for the chronic stage of psoriatic inflammation, making them a primary target for drug development [20]. In our study, we observed a significant level of inflammatory cells infiltrating human psoriatic skin biopsies, as well as increased expression of multiple pro-inflammatory cytokines in the lesion skins of psoriatic mice, which fully supports the notion that inflammatory cellular infiltration induced an inflammatory response that resulted in the pathogenesis of psoriatic skin lesions [21]. Multiple types of pro-inflammatory cytokines and chemokines have been linked with the pathological development of psoriasis, including Th1 cell-related cytokines IFN-γ and IL-12; Th17 cells associated cytokines IL-17, IL-22, and IL-23; and other pro-inflammatory cytokines such as IL-1β, IL-6, and TNF-α that promote the acute stage of inflammatory responses, or the chemokines that regulate neutrophil chemotaxis, such as IL-8/CXCL8. Similarly, we observed a significant reduction in the expression of IL-1β, IL-6, and TNF-α in the lesion skins of psoriatic mice treated with the α7nAchR agonist PNU-282987. These findings indicate that administration of α7nAchR agonist inhibits TH1 and TH17 differentiation and down-regulates pro-inflammatory cytokine expression, which may explain the molecular pathological mechanism underlying the anti-inflammatory effects of α7nAChR activation.

The differentiation of Th17 cells relies on the positive feedback regulation of the STAT3 signaling pathway, and STAT3 has been demonstrated to control cell cycling and proliferation in keratinocytes. The aberrant STAT3 activation in mouse skin keratinocytes has been linked to psoriasis-like skin inflammation [13]. Consistent STAT3 activation has been found in virtually every cell type involved in disease initiation and maintenance. Most of the crucial cytokines that are involved in disease pathogenesis, including the IL-23/IL-17/IL-22 axis, are also controlled by the STAT3 signaling pathway. Apart from neutralizing antibodies, such as those against IL-17 or IL-23, various STAT3 signaling pathway inhibitors, such as Tofacitinib, an oral JAK1/3 inhibitor [22], or Ruxolitinib, a topically applied JAK1/2 inhibitor that could block STAT3 phosphorylation, have been approved for clinical trials for the treatment of psoriasis [23, 24]. In line with previous studies [25, 26], our findings showed that STAT3 activation was significantly upregulated in the lesion skins of psoriatic mouse models, which was remarkably blocked in response to systematic in vivo PNU-282987 treatment. This result indicated that, in contrast to other parasitic biological therapies reagents such as antibodies against IL-17 (e.g. Brodalumab) [27] or IL-23 [28], which function by inhibiting the interaction between Th17 cytokines and their receptors, the anti-inflammatory effects of α7nAChR agonist PNU-282987 were mediated by its inhibitory effects on STAT3 signaling pathway activation, which resulted in a reduction of Th17-mediated inflammatory immune responses. To further confirm these findings, we measured the Th17-related cytokines involved in keratinocyte proliferation and differentiation, IL-6 and IL-22, which were found to be elevated in the blood of psoriasis patients and have been implicated in regulating keratinocyte proliferation and differentiation [29, 30]. Our findings further established that the anti-psoriatic effects of α7nAChR agonist PNU-282987 are mediated by inhibiting the STAT3 signaling pathway, as abnormal keratinocyte differentiation and proliferation were significantly alleviated in response to α7nAChR agonist PNU-282987 treatment. In contrast, administration of the α7nAChR antagonist Methyllycaconitine citrate exacerbated the disease by activating the STAT3 signaling pathway, increasing the abnormal proliferation and differentiation of skin keratinocytes in a psoriatic mouse model, indicating that α7nAChR activation is indeed providing critical immune protection in psoriasis-induced cutaneous inflammation.

NF-κB is another transcription factor that plays a role in the pathogenesis of psoriasis [31], and increased NF-κB activation has been observed in lesioned psoriatic skin [32]. Consistent with previous reports, we found constitutively activated NF-κB in the epidermis of experimental psoriasis. Given that NF-κB is a critical regulator of keratinocyte differentiation and proliferation, we next assessed whether the α7nAChR agonist PNU-282987 could modulate NF-κB activation. TNF-α, as an NF-kB upstream regulator, is one of the most important pro-inflammatory cytokines involved in acute stage inflammatory responses and has also emerged as a key mediator for the pathogenesis development of psoriasis [33], so we focused on this mediator and investigated the protective effects of PNU-282987 on TNF-α-induced inflammatory signaling pathway activation in keratinocytes. PNU-282987 effectively inhibited TNF induction of multiple types of pro-inflammatory cytokines, including IL-1β, IL-6, IL-8, and S100A9 mRNA expression in HaCaT cells. Furthermore, the TNFα-induced downregulation of Involucrin and upregulation of K17, both of which are associated with abnormal keratinocyte differentiation, were significantly reversed by the α7nAChR agonist PNU-282987 treatment. Notably, the biological effects of PNU-282987 were detectable in HaCaT cells at very low concentrations (0.1 ng/mL) and its anti-inflammation effects were obviously dose dependent. The dose kinetics of PNU-282987’s suppressive effect on TNF-α-induced inflammatory cytokine production indicated that a receptor-mediated action was very likely involved, as TNF receptor type 1 (p65) has been shown to be a major mediator of TNF-α-induced skin inflammation [34]. In subsequent mechanistic studies, we demonstrated that PNU-282987 is primarily targeting the canonical NF‐κB signaling pathway in response to TNF-α stimulation. PNU-282987 treatment significantly inhibited TNF-α-induced NF‐κB activation in vitro, primarily by reducing p65 nuclear accumulation and IκBα‐degradation.

In conclusion, our results suggest that using the α7nAchR agonist PNU-282987 to activate the CAP can reduce psoriasis-like skin lesions, which is likely mediated by its anti-proliferation and anti-inflammation effects. Methyllycaconitine citrate, on the other hand, may aggravate the pathological features of psoriasis, such as abnormal proliferation and differentiation. This study provides direct experimental evidence that PNU-282987 can reduce psoriatic inflammation by inhibiting the activation of the STAT3 and NF-κB signal pathways, thereby inhibiting Th17-related immune responses in psoriasis. More research is needed to fully understand the molecular pathological mechanism of PNU-282987 in the immune response and skin symptoms associated with psoriasis.

## Materials and methods

### Reagents

PNU-282987(HY-12560A, Med Chem Express) and Methyllycaconitine citrate (HY-N2332A, Med Chem Express) were purchased from Med Chem Express. Antibodies were as following, anti-Keratin 17 (ab53707, Abcam, Cambridge, UK), anti-Keratin 16 (sc-53255, Santa Crusz), anti-Keratin 10 (AF1861, Beyotime), anti-Phospho-STAT3 (Tyr705) (#9145, Cell Signaling Technology, Danvers, MA, USA), anti-Nicotinic Acetylcholine Receptor alpha 7 (ab216485, Abcam, Cambridge, UK), anti-Phospho-STAT3 (Tyr705)(#9145, Cell Signaling Technology, MA, USA), anti-STAT3 (#12640, Cell Signaling Technology, MA, USA), anti-Phospho-NF-κB p65 (#3033, Cell Signaling Technology, MA, USA), anti-NF-κB p65 (#8242, Cell Signaling Technology, MA, USA), anti-Phospho-IκBα (#2859, Cell Signaling Technology, MA, USA), anti-IκBα (#4812, Cell Signaling Technology, MA, USA) and GAPDH (BS60630, Bioworld technology Inc, MN, USA).

### Assessment of gene expression using publicly available datasets

We investigated the RNA and protein expression of α7nAChR in different human tissues on The Human Protein Atlas portal (Website: http://www.proteinatlas.org/). All data are available directly online.

### Patient biopsy skin samples

Biopsy skin samples were collected from five newly diagnosed psoriasis patients and five healthy volunteers undergoing esthetic plastic surgery. The patients were diagnosed based on clinical and histological examinations, and had not received any systemic or topical therapy for at least 2 weeks before skin biopsies were performed. The study protocol was approved by the ethics review board of the First Affiliated Hospital of Nanjing Medical University. Written informed consent was obtained from all participants prior to the study. All the patients and volunteers agreed with the publication of their’s photos.

### Animal models

Six to eight-week-old female BALB/c mice were purchased from the Model Animal Research Center of Nanjing University. The experimental procedures performed on mice were carried out according to the guidelines approved by the Nanjing Medical University Animal Welfare and Ethics committee. All of the animal experiment allocation and/or the outcome assessing were blinded performed. The following groups of mice were randomly assigned into cages and divided into four groups in the study, which included control group (mice received the treatment of vaseline cream), IMQ group (IMQ-induced psoriasis mouse model), IMQ + PNU group (psoriasis mouse received PNU-282987 treatment), PNU group (mice received PNU-282987 treatment), IMQ + METH group (psoriasis mouse received Methyllycaconitine citrate treatment) and METH group (mice received Methyllycaconitine citrate treatment). The IMQ-induced psoriasis mouse model was established following the protocol reported in the literature, briefly, female BALB/c mice received a daily topical application of IMQ cream (62.5 mg, Aldara 5%; 3 M Pharmaceuticals) on their shaved backs for 6 days to induce psoriasis-like skin, as described previously [35]. In the mice of the control group, vaseline was used in the same amount. To assess the therapeutic effects of α7nAChR activation in the psoriatic mouse model, IMQ-induced psoriatic mice were intraperitoneally injected with PNU-282987 (Med Chem Express, Shanghai, China, 10 mg/kg), or Methyllycaconitine citrate (Med Chem Express, Shanghai, China, 3 mg/kg), a specific agonist or antagonist of α7nAchR. 24 h after the last drug administration, the severity of cutaneous inflammation of the back skin was scored blindly by three independent investigators based on the clinical Psoriasis Area and Severity Index (PASI). The cumulative score represented the overall severity of inflammation (scale 0–9). Mice were euthanized by cervical dislocation and skin samples were taken from the treated dorsal skin and fixed in formalin for histological study or stored at −80°C for subsequent homogenization for western blot or qPCR assay.

### Cell culture

Cultured immortalized human keratinocyte (HaCaT) cells (NE Fusenig, Heidelberg, Germany) were obtained from KeyGen Biotech Company. The cells were cultured at 37 ˚C, 5% CO_2_ in Dulbecco’s Modified Eagle Medium (DMEM; Gibco; Thermo Fisher Scientific, Inc.) containing 10% fetal bovine serum (FBS; HyClone; Thermo Fisher Scientific, Inc.) and 100 units/mL penicillin, and 100 μg/mL streptomycin (Invitrogen; Thermo Fisher Scientific, Inc). The human monocytic THP-1 cell line was cultured in RPMI-1640 medium supplemented with 10% FBS and antibiotics at 37 ˚C, 5% CO_2_. HaCaT cells in good growth condition were seeded in a 6-well plate at 5 × 10^5^ cells/well. When 70% confluence was achieved, HaCaT cells were replaced with serum-free medium and starved overnight. The next day, different concentrations of PNU-282987 (0.1, 0.5, 1 uM) were supplemented and then the corresponding cytokine TNF-α (20 ng/ml) or IL -6 (25 ng/ml) or IL-22 (25 ng/ml) was added for 0.5 or 12 or 24 h. Total cell protein or total cell RNA was extracted for subsequent experiments.

### Histopathological and immunohistochemical staining

The skin specimens were fixed with 4% paraformaldehyde and embedded in paraffin. Serial sections were prepared and H&E, PAS, and Masson’s staining were performed following the instruction given in the manual AFIP Laboratory Methods in Histotechnology [36]. The immunostaining was performed using the streptavidin–peroxidase method. Anti-Nicotinic Acetylcholine Receptor alpha 7 (ab216485, Abcam, Cambridge, UK), anti-Ki67 (ab16667, Abcam, Cambridge, UK), anti-Keratin 17 (ab53707, Abcam, Cambridge, UK) and anti-Phospho-STAT3 (Tyr705) (Cell Signaling Technology #9145, Danvers, MA, USA) were used as primary antibodies.

### Western blot

Whole cell lysates were prepared from tissue homogenates and cells, separated on 10% SDS-polyacrylamide gel, and transferred to PVDF membranes. The membranes were incubated with 5% bovine serum albumin for 1 h and incubated with primary antibodies overnight at 4 °C. The following antibodies were used: anti-Nicotinic Acetylcholine Receptor alpha 7 (ab216485, Abcam, Cambridge, UK), anti-Phospho-STAT3 (Tyr705)(#9145, Cell Signaling Technology, MA, USA), anti-STAT3 (#12640, Cell Signaling Technology, MA, USA), anti-Phospho-NF-κB p65 (#3033, Cell Signaling Technology, MA, USA), anti-NF-κB p65 (#8242, Cell Signaling Technology, MA, USA), anti-Phospho-IκBα (#2859, Cell Signaling Technology, MA, USA), anti-IκBα (#4812, Cell Signaling Technology, MA, USA) and GAPDH (BS60630, Bioworld technology Inc, MN, USA). Blots were incubated with HRP-conjugated secondary antibodies (BS13278, Bioworld technology Inc, MN, USA) for 1 h, and protein expression was detected with ECL (WBULS0500, Millipore, MA, USA) by digital imaging systems (SYNGENE, Cambridge, UK).

### Quantitative reverse transcription polymerase chain reaction

Total RNA was isolated from tissues or cells using TRIzol reagent (Sigma, Milwaukee, WI, USA) and 1 μg of total RNA was used for reverse transcription reaction via the PrimeScript RT Master Kit (Takara Kyoto, Japan) to synthesize cDNA. Primers are listed in the Table. All quantitative polymerase chain reaction assays were performed in the QuantStudio 5 Real-Time PCR system (Applied Biosystems, CA, USA) with Power-Up SYBR Master Mix (Applied Biosystems, CA, USA) under the following conditions: 95 °C for 15 min, and 95 °C for 15 s, 60 °C for 1 min, for 40 cycles. β-actin or GAPDH was used as the internal control. The relative change in the levels of genes of interest was determined by the 2^ΔΔCT^ method.

### Statistical analysis

Statistical analyses were performed using GraphPad Prism software 8.0 (GraphPad Inc., San Diego, CA). All the results were shown as mean ± SD. If data had a normal distribution, Student *t*-test or One-way ANOVA was used to test differences in quantitative data among different groups. Otherwise, the statistical difference was calculated by the Wilcoxon test or Kruskal–Wallis *H*-test. Two-side test was performed. Differences with *P* < 0.05 were considered statistically significant.

## Supplementary information

original western blots

## Data Availability

All data included in this study are available upon request by contact with the corresponding author.
