# Supplementary material for: Alpha-7 nicotinic acetylcholine receptor agonist alleviates psoriasis-like inflammation through inhibition of the STAT3 and NF-κB signaling pathway
Source: Cell Death Discov. 2022 Mar 30;8:141. doi: 10.1038/s41420-022-00943-4 (PMC8964744; doi:10.1038/s41420-022-00943-4)
Supplement: Supplementary file 1 — original western blots [file 41420_2022_943_MOESM1_ESM.pdf]

The original western blots were listed as following.

Figure1 b

α7nAchR

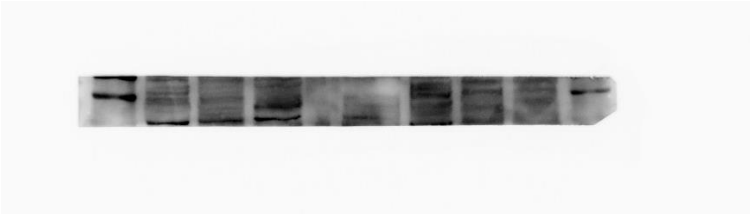

GAPDH

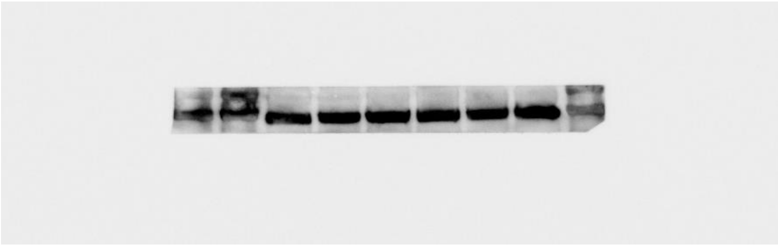

1 c

α7nAchR

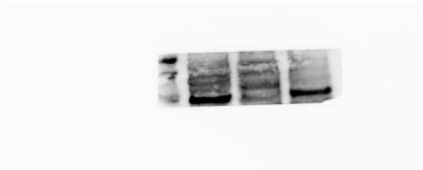

GAPDH

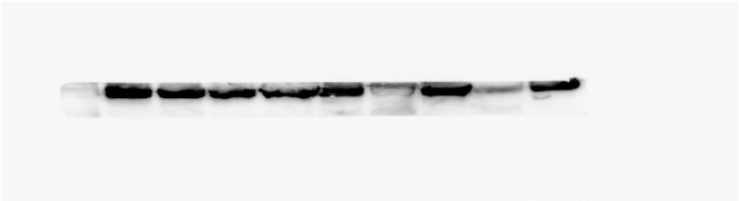

1e

α7nAchR

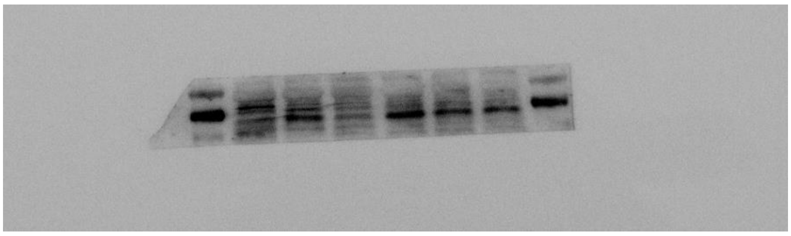

GAPDH

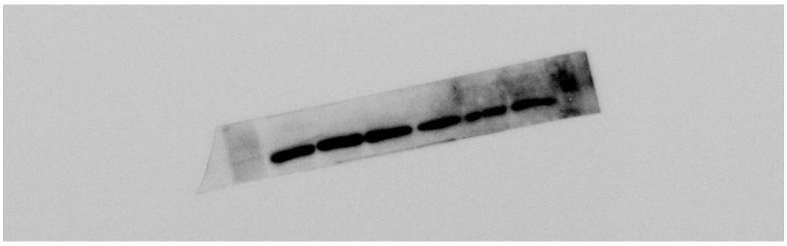

Figure3

3b

P-STAT3

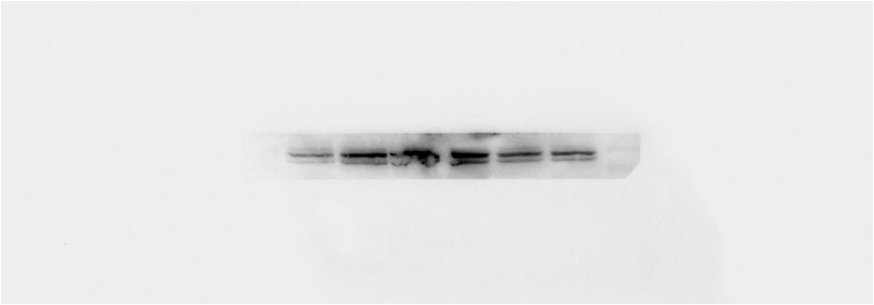

STAT3

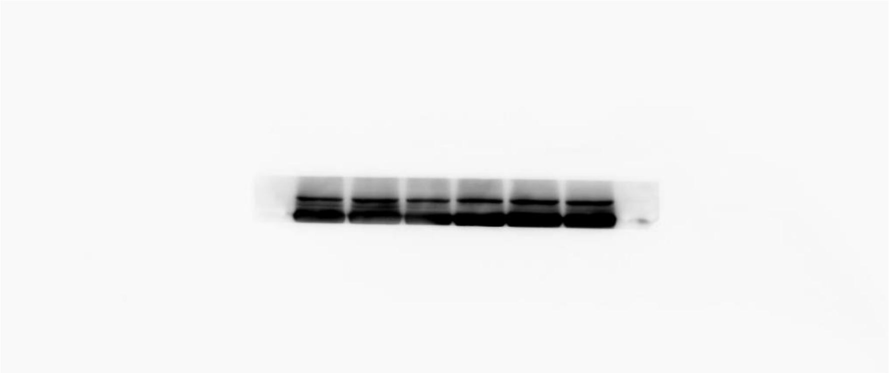

3d

IL-1β

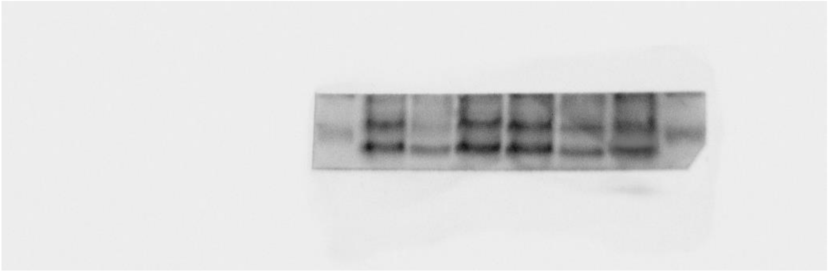

GAPDH

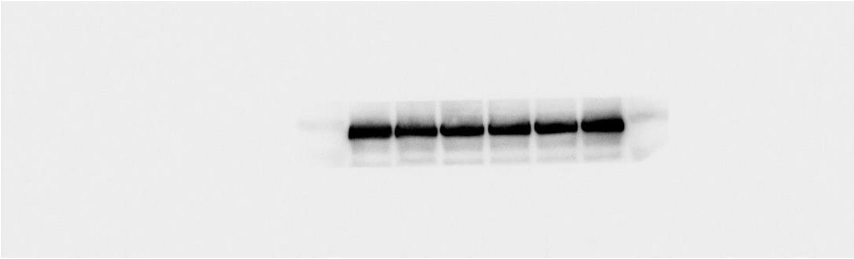

Figure 4

4a

Ki67

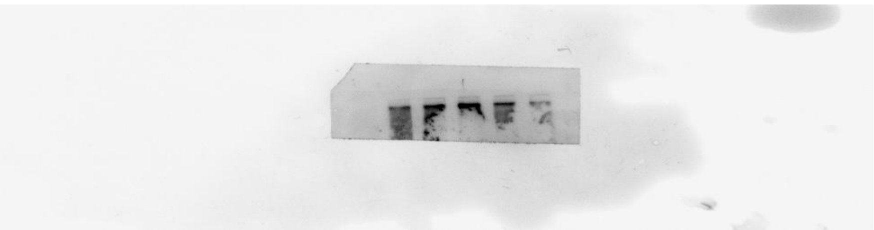

K16

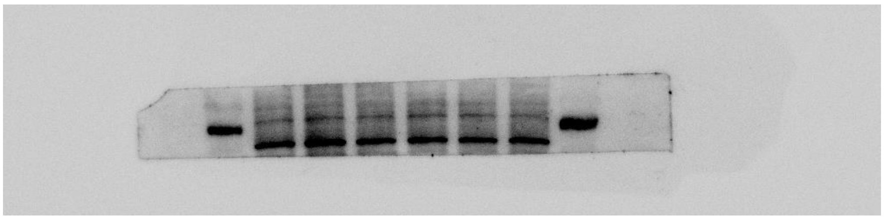

K17

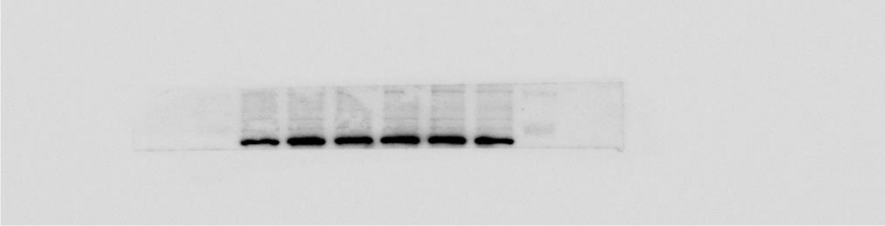

P-STAT3

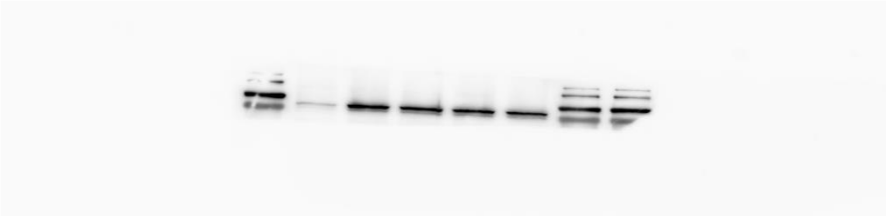

STAT3

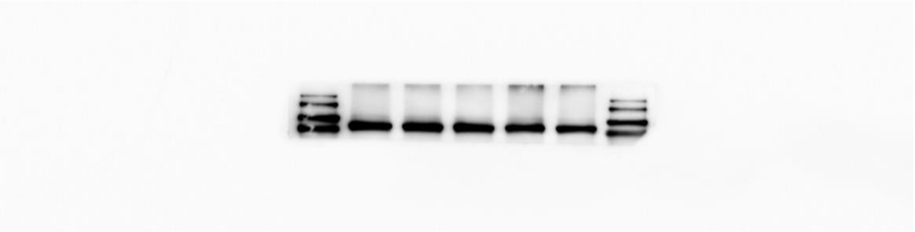

GAPDH

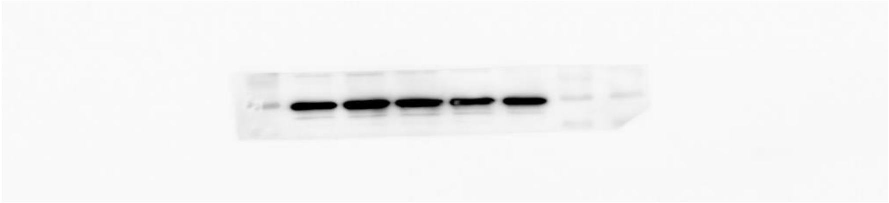

4c

K16

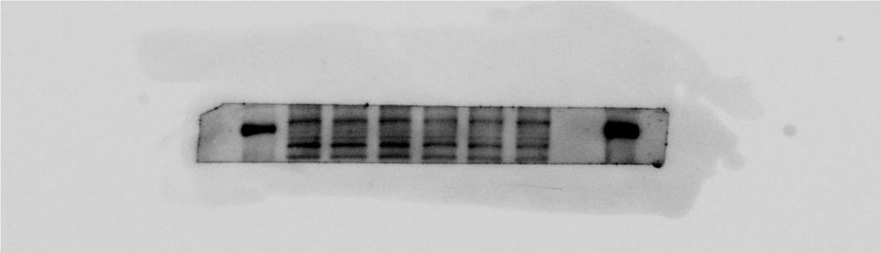

K17

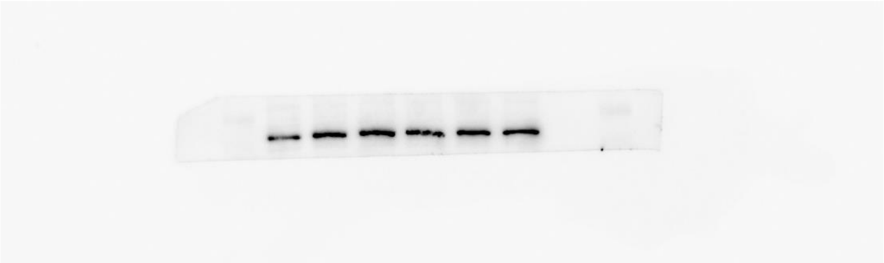

P-STAT3

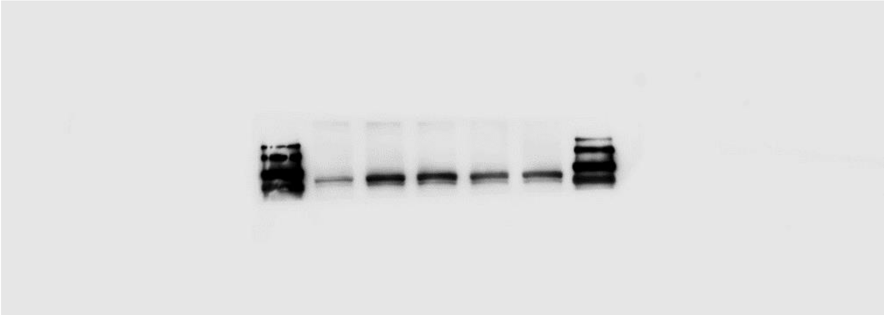

STAT3

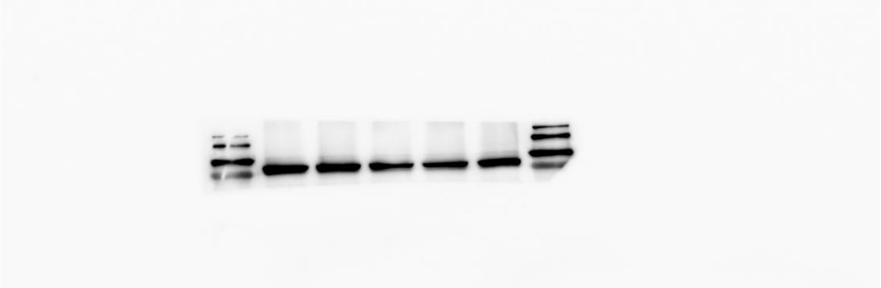

GAPDH

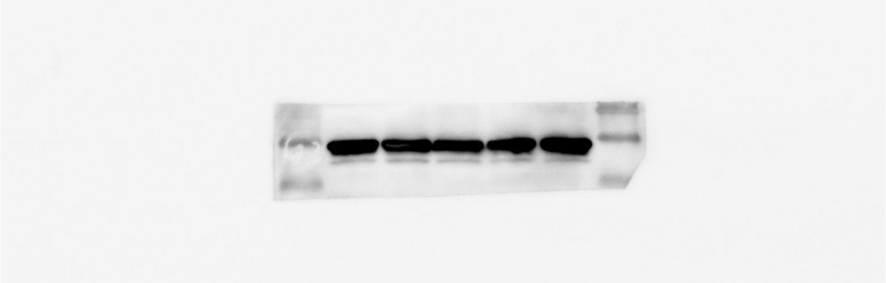

Figure 6

6b

P-IKBα

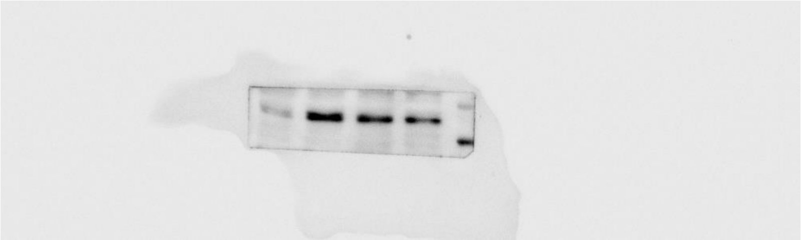

IKBα

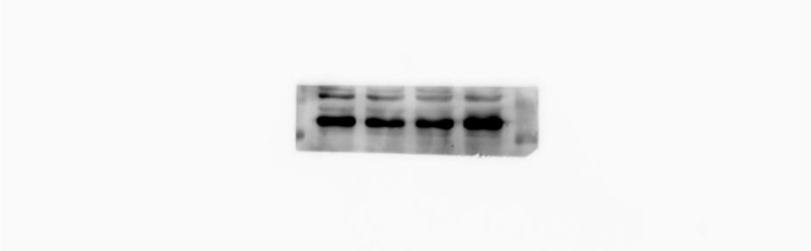

P-P65

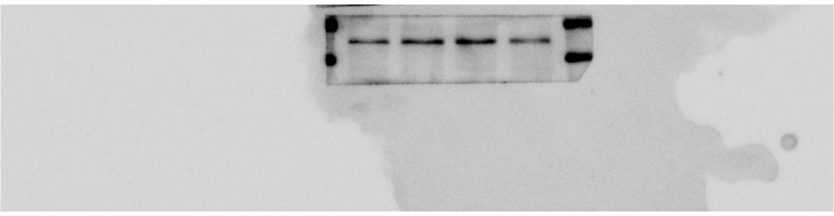

P65

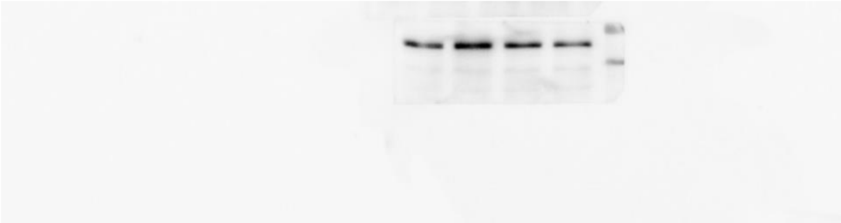

GAPDH

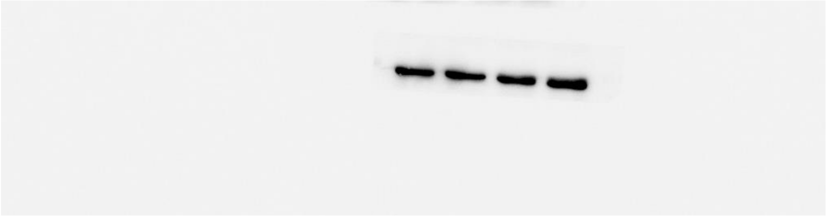

Figure 8

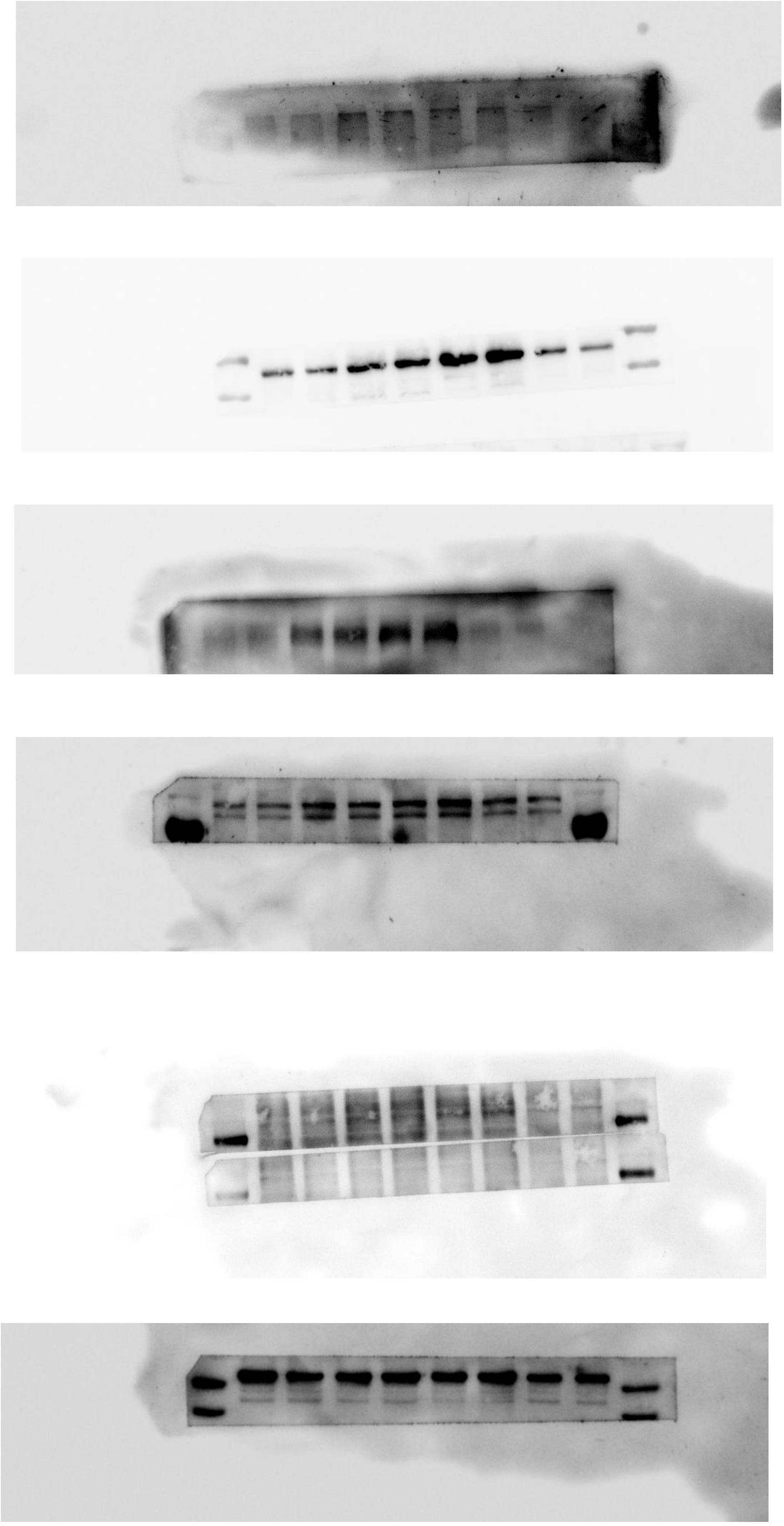

Ki67

K17

K16

P-STAT3

STAT3

GAPDH
